# A complex with poly(A)-binding protein and EWS facilitates the transcriptional function of oncogenic ETS transcription factors in prostate cells

**DOI:** 10.1016/j.jbc.2023.105453

**Published:** 2023-11-11

**Authors:** Benjamin M. Greulich, Saranya Rajendran, Nicholas F. Downing, Taylor R. Nicholas, Peter C. Hollenhorst

**Affiliations:** 1 Department of Biology, Mercer University, Macon, Georgia, USA; 2 Medical Sciences Program, Indiana University School of Medicine, Bloomington, Indiana, USA

**Keywords:** ERG, prostate cancer, EWS, PABPC1, transcription, ETS factor

## Abstract

The ETS transcription factor ERG is aberrantly expressed in approximately 50% of prostate tumors due to chromosomal rearrangements such as *TMPRSS2/ERG*. The ability of ERG to drive oncogenesis in prostate epithelial cells requires interaction with distinct coactivators, such as the RNA-binding protein EWS. Here, we find that ERG has both direct and indirect interactions with EWS, and the indirect interaction is mediated by the poly-A RNA-binding protein PABPC1. PABPC1 directly bound both ERG and EWS. ERG expression in prostate cells promoted PABPC1 localization to the nucleus and recruited PABPC1 to ERG/EWS-binding sites in the genome. Knockdown of PABPC1 in prostate cells abrogated ERG-mediated phenotypes and decreased the ability of ERG to activate transcription. These findings define a complex including ERG and the RNA-binding proteins EWS and PABPC1 that represents a potential therapeutic target for ERG-positive prostate cancer and identify a novel nuclear role for PABPC1.

Prostate cancer has a high prevalence of chromosomal rearrangements that fuse highly active, and often androgen-driven, promoters to the ORFs of ETS family transcription factors. This leads to transcriptional activation and expression of ETS factors that are not expressed in normal prostate epithelia. These gene fusions tend to be mutually exclusive, with the most common fusion occurring between the androgen-driven promoter of *TMPRSS2* and the ETS factor *ERG* (1, 2). The *TMPRSS2-ERG* fusion is present in approximately 50% of prostate cancers, while fusions with ETS genes *ETV1*, *ETV4*, and *ETV5* occur in approximately 10%, 2 to 5%, and 1% of cases, respectively (3, 4). Expression of these ETS genes can drive oncogenic phenotypes such as migration and clonogenic survival in prostate cell lines, and when combined with "second hit" mutations these ETS factors can drive tumor growth (3, 5, 6, 7). Due to the high prevalence of ETS transcription factor deregulation in prostate cancer, they are attractive therapeutic targets. One strategy to target ETS transcription factors, which lack enzymatic- and ligand-binding domains, is to disrupt protein–protein interactions. For this reason, it is important to define protein complexes critical for oncogenic ETS transcription factor function in prostate cells.

One critical interacting partner of oncogenic ETS proteins is the RNA-binding protein EWS. We have previously demonstrated that EWS interacts with ERG, ETV1, ETV4, and ETV5, but no other ETS family transcription factors. EWS directly interacts with ERG, ETV4, and ETV5, while the interaction between EWS and ETV1 is indirect (8). This EWS interaction is required for the ability of ERG to activate of target genes and drive tumorigenesis. EWS is a ubiquitously expressed FET family protein that contains an intrinsically disordered prion-like domain in its N terminus and multiple nucleic acid binding domains in its C terminus (9, 10, 11). EWS can interact with RNA polymerase II to regulate gene transcription (12, 13) and also plays roles in splicing and response to DNA damage (14, 15). The intrinsically disordered N terminus of EWS (ntEWS), and other FET proteins, can facilitate the generation of membraneless condensates, which have been tied to regulation of gene expression (16, 17, 18). This EWS N terminus is also fused to the C terminus of ETS family transcription factors in the protein products of the chromosomal translocations that drive Ewing sarcoma. Within these fusion proteins, the EWS N terminus acts as a strong transcriptional activation domain (9, 19, 20, 21).

PABPC1 is a ubiquitously expressed member of a family of poly(A) RNA–binding proteins that generally localizes to the cytoplasm. Other members of the family include the nuclear PABPN1 and the tissue-specific proteins PABPC2, PABPL1, PABP4, and the less understood PABPC5 (22, 23). While PABPC1 is predominantly cytoplasmic, it can shuttle between the nucleus and cytoplasm to facilitate the export of nascently transcribed mRNAs (24, 25). In the cytoplasm, PABPC1 is a marker of cytoplasmic stress granules, where it regulates mRNA stability and mediates nonsense-mediated decay through interactions mediated by its four RNA recognition motifs (22, 26).

Here, we found that, in addition to the direct interaction between ERG and the ntEWS, there was also an indirect interaction between ERG and the ctEWS. Immunoprecipitation (IP) coupled with mass spectrometry identified PABPC1 as a possible interacting partner of both ERG and ctEWS. While purified ERG and ctEWS failed to interact, the addition of purified PABPC1 promoted complex formation. Furthermore, knockdown of PABPC1 reduced the interaction between ERG and EWS in prostate cancer cells. ERG expression drove nuclear localization and chromatin binding of PABPC1, and PABPC1 knockdown attenuated ERG-driven migration, clonogenic survival, and transcriptional activation. Taken together, these data indicate that a complex between ERG, EWS, and PABPC1 is necessary for ERG activity in prostate cells and represents a novel therapeutic target.

## Results

### ERG interacts with both ntEWS and ctEWS

**Figure 1:**
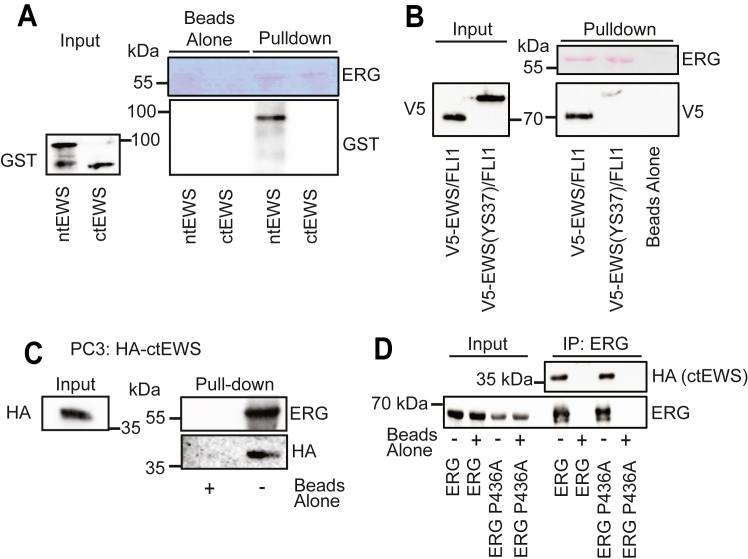
**ERG participates in two distinct interactions with EWS.***A*, affinity pulldown of purified GST-tagged ntEWS (1–355) and ctEWS (356–656) using purified ERG bound to magnetic beads. *B*, affinity pulldown of V5-tagged EWS/FLI1 and mutant EWS(YS37)/FLI1 expressed in PC3 cells using purified ERG bound to magnetic beads. *C*, immunoblots of affinity pulldown of hemagglutinin-tagged ctEWS exogenously expressed in PC3 whole-cell extracts using purified ERG bound to magnetic beads. *D*, immunoblot of coimmunoprecipitated WT ERG and mutant ERG P436A expressed in PC3-ctEWS whole-cell extracts. ctEWS, C terminus of EWS; GST, glutathione-*S*-transferase.

Glutathione-*S*-transferase (GST)-EWS 1 to 355 (ntEWS) and GST-EWS 356 to 656 (ctEWS) were purified and tested for binding to purified ERG. Consistent with our previous finding (27), only ntEWS bound ERG directly (Fig. 1*A*). Ewing sarcoma is driven by chromosomal rearrangements that create fusion proteins consisting of the ntEWS and the C-terminus of an ETS family transcription factor such as FLI1 or ERG (28, 29). As this ntEWS can interact with ERG (Fig. 1*A*), we tested EWS/FLI1 for ERG binding. As predicted, V5-tagged EWS/FLI1 expressed in PC3 prostate cancer cells bound to purified ERG (Fig. 1*B*). Mutation of 37 tyrosines to serine (YS37) in the EWS N terminus disrupts EWS/FLI1 phase separation (16). This mutation also diminished the interaction with ERG (Fig. 1*B*), suggesting a possible connection between the ERG interaction and the phase-separation function of EWS.

As a further test of the ERG/EWS interaction, the ctEWS was expressed in PC3 cells and cell extracts were exposed to purified ERG bound to beads. Surprisingly, ctEWS in PC3 whole-cell extracts bound purified ERG (Fig. 1*C*). This suggested that, in addition to directly interacting with the ntEWS, ERG has a second indirect interaction with ctEWS. A P436A mutation of ERG disrupts the direct interaction of ERG with EWS (8), however this mutation did not diminish the indirect interaction between ERG and ctEWS (Fig. 1*D*). This provides further evidence that ERG participates in two distinct interactions with EWS.

### PABPC1 is an interacting partner of ctEWS and oncogenic ETS factors

**Figure 2:**
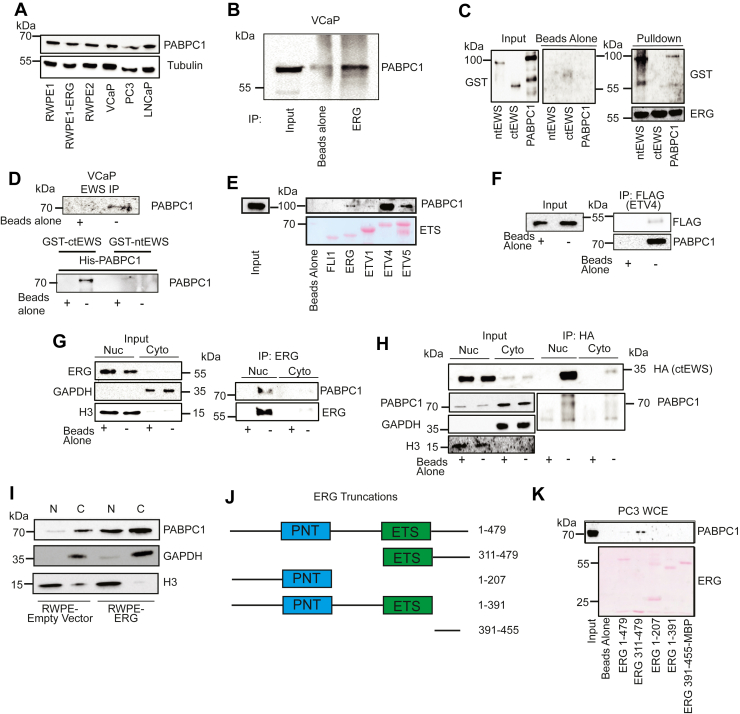
**PABPC1 is an interacting partner of ERG and ctEWS.***A*, immunoblot of PABPC1 in various prostate cell lines. *B*, immunoprecipitation with anti-ERG antibody, immunoblotted for PABPC1. *C*, immunoblot of affinity pulldown experiments of purified GST-tagged EWS fragments or PABPC1 using purified His-ERG bound to magnetic beads. *D*, immunoprecipitation from VCaP lysate with anti-EWS, immunoblotted for PABPC1 (*top*) and pulldown of purified PABPC1 by either purified ctEWS or ntEWS bound to magnetic beads (*bottom*). *E*, immunoblot of an affinity pulldown of PABPC1 using purified oncogenic ETS factors (ERG, ETV1, ETV4, and ETV5) and the nononcogenic FLI1. Purified protein quantity was measured by Ponceau staining (*lower*). *F*, immunoprecipitation of FLAG-tagged ETV4 from RWPE cells, immunoblotted for endogenous PABPC1. *G*, immunoblot of coimmunoprecipitation of endogenous ERG and PABPC1 in nuclear and cytoplasmic fractions of RWPE-ERG cells. *H*, immunoblot of coimmunoprecipitation of hemagglutinin-tagged ctEWS in nuclear and cytoplasmic fractions of PC3-ctEWS cells. *I*, immunoblot of PABPC1 in nuclear and cytoplasmic fractions of RWPE-empty vector or RWPE-ERG cells. H3 and GAPDH are nuclear (N) and cytoplasmic (C) markers, respectively. *J*, diagrams of ERG truncations show locations of pointed (PNT) and ETS DNA-binding domains (ETS). *K*, immunoblot of affinity pulldown of PABPC1 from PC3 nuclear extracts using the purified ERG fragments shown in (*J*) bound to magnetic beads. ERG 392 to 455 only is also fused to maltose-binding protein (MBP). Purified ERG protein quantity was visualized by Ponceau staining. ctEWS, C terminus of EWS; GST, glutathione-*S*-transferase; PABPC1, poly-adenylate–binding protein.

Both direct and indirect interactions between ERG and EWS suggest that these proteins are in a complex with one or more additional proteins. To determine what protein or proteins are necessary for facilitating the indirect interaction between ctEWS and ERG, ctEWS was immunoprecipitated from PC3 cells expressing both ctEWS and ERG and analyzed *via* mass spectrometry. After filtering out probable contaminants, the potential interacting partners were cross-referenced with published ERG IP-mass spectrometry experiments (30). One protein that was found bound to both ERG and ctEWS was cytoplasmic poly-adenylate–binding protein 1 (PABPC1). PABPC1 was ubiquitously expressed across various prostate cell lines, including androgen receptor (AR)-negative RWPE and PC3 cells as well as AR-positive VCaP and LNCaP cells (Fig. 2*A*). Co-IP of PABPC1 using an anti-ERG antibody in TMPRSS2/ERG-positive VCaP prostate cancer cells demonstrated that endogenous ERG-bound PABPC1 (Fig. 2*B*). Purified ERG bound to nickel beads pulled down purified GST-ntEWS and GST-PABPC1, but not GST-ctEWS (Fig. 2*C*). Thus, there is a direct interaction between ERG and PABPC1. Co-IP of PABPC1 with an EWS antibody demonstrated that endogenous EWS-bound PABPC1 in VCaP cells (Fig. 2*D* top). Purified GST-ctEWS, but not purified GST-ntEWS, pulled down purified His-PABPC1 (Fig. 2*D* bottom), indicating the interaction between EWS and PABPC1 is direct and occurs in the EWS C terminus.

Four ETS family transcription factors (ETV1, ETV4, ETV5, and ERG) can interact with EWS, whereas the ERG homolog FLI1 cannot (8). The interaction between EWS and ERG, ETV4, and ETV5 is direct, but the interaction between EWS and ETV1 is indirect (8). Purified ETS factors were used to test the specificity of PABPC1 binding. Similar to EWS, PABPC1 directly bound ERG, ETV4, and ETV5, but not ETV1 or FLI1 (Fig. 2*E*). Co-IP verified the interaction between ETV4 and PABPC1 in extracts of RWPE1 cells–expressing Flag-ETV4 (Fig. 2*F*).

Interactions between PABPC1 and nuclear proteins such as ERG, ETV4, and EWS are somewhat unexpected, as PABPC1 has been reported as a cytoplasmic protein (22, 31, 32), and is even named as such. To test where an interaction occurs in cells, ERG was immunoprecipitated from nuclear and cytoplasmic fractions of RWPE-ERG lysates (Fig. 2*G*). PABPC1 co-immunoprecipitated with ERG exclusively in the nuclear fraction. Nuclear and cytoplasmic fractionation followed by co-IP experiments were also used to verify a nuclear ctEWS–PABPC1 interaction in PC3 cells expressing hemagglutinin (HA)-ctEWS (Fig. 2*H*). To determine if PABPC1 is present in the nucleus of prostate cells, PABPC1 protein expression was determined in nuclear and cytoplasmic fractions of RWPE-empty vector and RWPE-ERG cells (Fig. 2*I*). Consistent with previous findings (22, 32), PABPC1 was almost exclusively cytoplasmic in RWPE-empty vector cells. Surprisingly, PABPC1 was equally distributed between the nucleus and cytoplasm in RWPE-ERG cells. Immunofluorescence imaging of PABPC1 in RWPE1 and RWPE-ERG cells shows a similar redistribution, with PABPC1 present in the nucleus when ERG is expressed (Fig. S1). This shift in localization upon ERG expression helps explain the ability of ERG to bind PABPC1 in the nucleus. To identify the region of ERG that interacts with PABPC1, purified ERG fragments (Fig. 2*J*) were bound to beads and mixed with PC3 whole-cell extract. As expected, full-length ERG bound to PABPC1 (Fig. 2*K*). The interaction disappeared for all fragments except for one (311–479) that contains both the ETS DNA-binding domain and the C terminus of the protein, suggesting that both of these regions are important for binding.

### PABPC1 mediates the interaction between ERG and ctEWS

**Figure 3:**
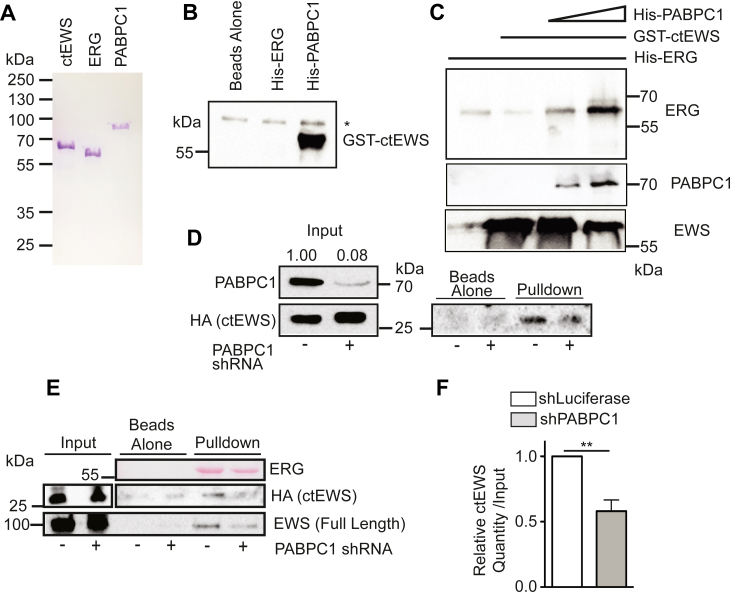
**PABPC1, ERG, and EWS form a complex.***A*, coomassie blue staining of purified GST-ctEWS, His-ERG, and His-PABPC1. *B*, anti-GST immunoblot of pulldown of GST-ctEWS (all lanes) with nickel beads in the absence or presence of His-ERG or His-PABPC1. *C*, anti-ERG Immunoblot of pulldown of His-ERG using glutathione beads in the presence or absence of GST-ctEWS, and with or without increasing concentrations of His-PABPC1. *D*, immunoblot of PABPC1 or hemagglutinin-ctEWS from VCaP cells–expressing hemagglutinin-ctEWS and shRNAs-targeting luciferase (−) or PABPC1 (+). Pull down is binding to nickel beads coated with purified His-ERG. Densitometry values normalized to VCaP-ctEWS plus luciferase-targeted shRNA are displayed. *E*, a replicate using same knockdown cells as shown in (*D*), but including loading control for ERG (Ponceau), and immunoblot for endogenous full-length EWS. *F*, densitometry quantification of ctEWS in pulldown from (*D*) and (*E*) plus an additional biological replicate (mean + SEM). ctEWS, C terminus of EWS; GST, glutathione-*S*-transferase; PABPC1, poly-adenylate–binding protein.

To further characterize the complex between ERG, PABPC1 and EWS, GST-ctEWS, His-ERG, and His-PABPC1 were purified (Fig. 3*A*). Nickel beads coated with His-PABPC1, but not His-ERG could pull-down GST-ctEWS (Fig. 3*B*). This direct interaction between PABPC1 and ctEWS and the direct interaction between PABPC1 and ERG (Fig. 2*B*), suggests that PABPC1 could mediate the indirect interaction between ERG and ctEWS. To test this, His-ERG was added to GSH beads. The addition of GST-ctEWS did not increase ERG binding compared to beads alone (Fig. 3*C*). However, the addition of His-PABPC1 and GST-ctEWS promoted binding of ERG, indicating a complex of the three proteins (Fig. 3*C*). To test if endogenous PABPC1 is necessary for the indirect interaction between ERG and ct-EWS, HA-ctEWS was expressed in VCaP prostate cancer cells, with or without PABPC1 shRNA knockdown, and cell extracts exposed to beads bound with purified His-ERG. Knockdown of PABPC1 decreased the interaction between ERG and ctEWS (Fig. 3*D*). To confirm this finding, the experiment was repeated, and a similar result was observed (Fig. 3*E*). Quantification of four biological replicates of the pull down found a significant decrease in the ERG/ctEWS interaction from cell extracts with PABPC1 knockdown (Fig. 3*F*). Interestingly, the interaction between ERG and endogenous full-length EWS also decreased with PABPC1 knockdown (Fig. 3*E*), indicating that this indirect interaction adds to the stability of the ERG/EWS complex.

### ERG promotes PABPC1 binding to chromatin

**Figure 4:**
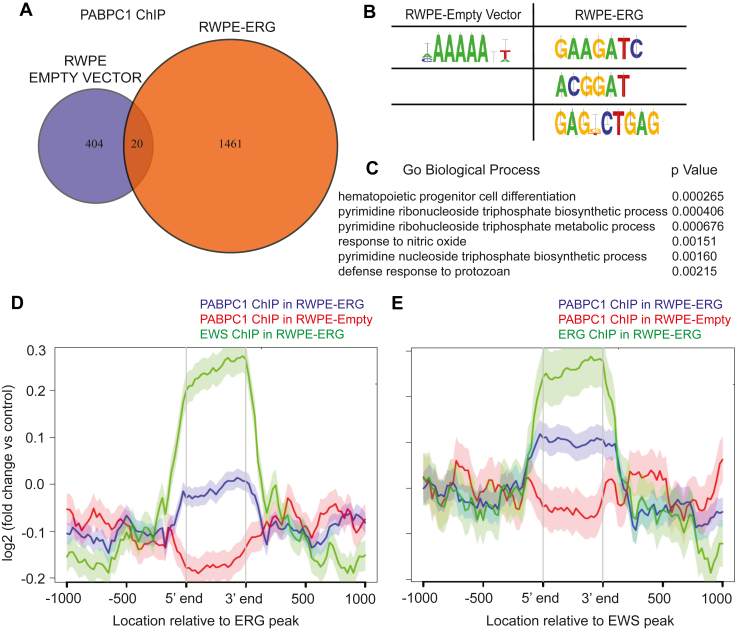
**ERG promotes PABPC1 binding to chromatin.***A*, overlap of PABPC1 ChIP-seq peaks in RWPE-empty vector and RWPE-ERG cells. *B*, most overrepresented motifs in PABPC1 bound sites. *C*, most overrepresented biological process ontologies of nearest neighbor genes to PABPC1 ChIP-seq called peaks in RWPE-ERG cells using ChIP-ENRICH. *D* and *E*, metagene plot of PABPC1 ChIP reads from RWPE-empty vector and RWPE-ERG cells compared to ERG and EWS ChIP reads centered on called ERG (E) or EWS (F) peaks. ChIP, chromatin immunoprecitation; PABPC1, poly-adenylate–binding protein.

While PABPC1 is typically observed in the cytoplasm, it has been reported to enter the nucleus, particularly with respect to its function as a nuclear-cytoplasmic shuttle (24). It can also bind nuclear mRNAs and promote nuclear retention (25, 33). However, relatively little is known regarding the function of nuclear PABPC1. The interactions described above between PABPC1 and ERG as well as the coactivator EWS suggest a potential role of PABPC1 in a chromatin-affiliated higher order complex. To determine if PABPC1 localizes to chromatin, chromatin immunoprecitation (ChIP)-seq of PABPC1 was performed in RWPE-empty vector and RWPE-ERG cells. 1481 PABPC1-bound peaks were called in RWPE-ERG cells, with only 424 peaks in RWPE-empty vector cells (Fig. 4*A*). Only 20 bound sites overlapped. This suggests that in the absence of ERG, PABPC1 participates in very little chromatin binding, but expression of ERG can promote PABPC1 localization to chromatin, consistent with the change in nuclear localization (Fig. 2*G*). In RWPE-empty vector cells, the most enriched motif in PABPC1-binding sites was 5′-AAAAA-3′ (Fig. 4*B*). It is not clear if this is related to the ability of PABPC1 to bind poly-A RNA. Enriched sequences in RWPE-ERG cells were different, and included an ETS motif, 5′-ACGGAT-3′. Overrepresented ontologies of nearest neighbor genes to PABPC1-binding sites were identified using ChIP-ENRICH (34, 35, 36, 37). The highest ranking ontology in the RWPE-ERG dataset was hematopoietic progenitor cell differentiation (Fig. 4*C*), which is interesting as this is a normal function of ERG (38). PABPC1 ChIP-seq was then compared to our previously published ChIP-seq of ERG and EWS in the same cell line (8). Aligned reads of PABPC1 and EWS ChIP-seq in RWPE-ERG cells were enriched at called ERG peaks, but reads from PABPC1 ChIP in RWPE-empty vector cells were not enriched (Fig. 4*D*). This trend was also observed when aligned reads were centered on called peaks from EWS ChIP in RWPE-ERG cells (Fig. 4*E*). Taken together, these data indicate that ERG can recruit PABPC1 to genomic sites bound by ERG and EWS.

### PABPC1 is important for biological functions of ERG in prostate cells

**Figure 5:**
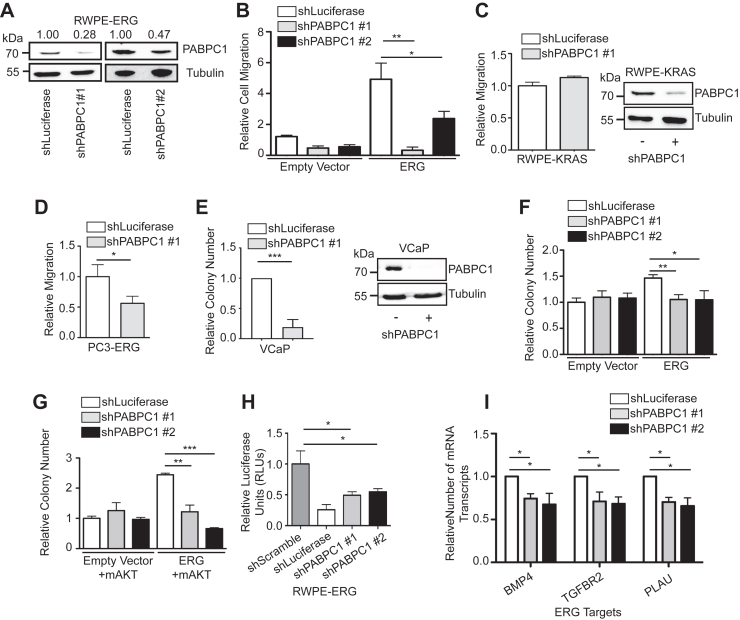
**PABPC1 is important for biological functions of ERG in prostate cells.** *A*, immunoblot of PABPC1 in RWPE cells–expressing ERG and shRNAs-targeting luciferase or PABPC1. Densitometry was performed to calculate the knockdown efficiency for each shRNA. Densitometry values normalized to RWPE-empty vector plus luciferase-targeted shRNA are displayed. *B*, *trans-*well migration assay of RWPE-empty vector and RWPE-ERG cells with PABPC1 or control luciferase targeted shRNAs. Migration was normalized to RWPE-empty vector cells with luciferase-targeted shRNA. *C*, *trans-*well migration assay of RWPE-KRAS (RWPE2) cells with PABPC1-targeted shRNA normalized to RWPE-KRAS plus luciferase-targeted shRNA. Immunoblot demonstrates PABPC1 knockdown. *D*, *trans-*well migration assay of PC3-ERG cells with PABPC1 shRNA normalized to PC3-ERG plus luciferase shRNA. *E*, relative number of colonies formed after low-density plating of VCaP cells with or without PABPC1 knockdown, shLuciferase is set to 1, n = 6. Immunoblot demonstrates PABPC1 knockdown. *F*, relative number of colonies formed following low-density plating of cell lines from (*B*). Colony number was normalized to RWPE-empty vector plus luciferase-targeted shRNA. *G*, relative number of colonies formed following low-density plating of cell lines from (*B*) with transient transfection of myristoylated AKT (mAKT). Colony number was normalized to RWPE-empty vector plus luciferase-targeted shRNA. *H*, luciferase assay of RWPE-empty vector and RWPE-ERG cells with or without PABPC1-targeted shRNAs. ERG activity was determined using a reporter containing seven consecutive repeats of the ETS-binding site GGAA. The mean and SEM of four biological replicates are plotted. *I*, RT-qPCR of three ERG target genes used in (39). Quantity of mRNA transcripts were first normalized to 18S, and then normalized to RWPE-ERG + shLuciferase. Unless otherwise indicated, all experiments are plotted as the mean and SEM of three biological replicates. *p*-values were calculated using Student’s *t* test, where ∗ indicates a *p*-value < 0.05, ∗∗ indicates a *p*-value < 0.01, and ∗∗∗ indicates a *p*-value < 0.001. GST, glutathione-*S*-transferase; PABPC1, poly-adenylate–binding protein; qPCR, quantitative PCR.

PABPC1 expression is significantly higher in prostate tumors than normal prostate tissue (Fig. S2*A*). To determine if PABPC1 is important for ERG function, we first used the RWPE1 cell line system, where we have previously demonstrated that ERG can drive tumorigenesis in cooperation with AKT activation (6, 8). Two independent shRNAs were used to knockdown PABPC1 (Fig. 5*A*). ERG expression significantly promoted RWPE1 cell migration in a *trans-*well assay, but this phenotype was attenuated with PABPC1 knockdown (Fig. 5*B*). This effect was specific to cells expressing ERG, as PABPC1 knockdown did not alter migration of RWPE cells that migrate due to KRAS overexpression and do not express ERG (Fig. 5*C*). We have previously shown that expression of ERG can promote migration of PC3 prostate cancer cells (39), and migration of PC3-ERG cells was also decreased by PABPC1 knockdown (Fig. 5*D*).

VCaP prostate cancer cells express ERG due to a TMRPSS2/ERG rearrangement. Knockdown of PABPC1 in VCaP cells decreased clonogenic growth (Figs. 5*E* and S2*B*). In the RWPE cell system, exogenous ERG weakly promotes clonogenic survival alone, but more strongly drives this phenotype when AKT signaling is active (6). PABPC1 knockdown reduced ERG’s ability to drive clonogenic survival both in a low AKT environment (Fig. 5*F*) and in the presence of constitutively active AKT (Fig. 5*G*) but had no effect on the clonogenic survival of RWPE-empty vector cells.

To test the role of PABPC1 on ERG transactivation, luciferase reporter assays were performed using a 7×GGAA repeat reporter that we previously demonstrated is activated by ERG in RWPE1 cells (8). Compared to a scramble sequence shRNA, the luciferase signal was sharply diminished by a luciferase-directed shRNA, validating the observed signal is due to the activity of luciferase (Fig. 5*H*). The luciferase activity also decreased in cells containing PABPC1 shRNAs, indicating PABPC1 is important for ERG’s ability to activate transcription. PABPC1 knockdown also resulted in reduced mRNA levels of previously validated (39) ERG target genes (Fig. 5*I*). A reduced level of RNA polymerase II was also observed in the body of ERG target genes by ChIP-quantitative PCR (qPCR), indicating reduced transcription when PABPC1 is knocked down (Fig. S3*A*). We and other groups have observed that ERG does not drive two-dimension cell proliferation *in vitro* (3, 7, 8, 40). Consistent with this, RWPE proliferation was statistically unchanged, regardless of ERG or PABPC1 status (Fig. 3, *B* and *C*). This finding also indicates that the changes in cell migration and clonogenic growth observed after PABPC1 knockdown were not due to reduced proliferation or cellular toxicity.

## Discussion

Here, we provide evidence that ERG functions in a complex with the RNA-binding proteins EWS and PABPC1. ERG expression shifted PABPC1 from the cytoplasm to the nucleus, and promoted binding to chromatin. Furthermore, upon ERG expression, PABPC1-bound sites changed from poly (A) motifs to new motifs, including the canonical ETS motif, 5′-GGA(A/T)-3′. Unique gene ontologies of PABPC1-bound genes in ERG-positive cells were associated with ERG function, including hematopoietic progenitor cell differentiation and ontologies relating to migration. To our knowledge, this represents the first evidence of PABPC1 binding to chromatin and functioning as a potential transcriptional coregulator. While the specific mechanism of how PABPC1 facilitates activation of ERG target gene expression remains unknown, we hypothesize PABPC1 stabilizes ERG’s interaction with EWS to recruit RNA polymerase II or other members of the transcription initiation complex.

Both PABPC1 and EWS are RNA-binding proteins. It is unclear if there is any role for RNA in regulating the oncogenic function of ETS transcription factors in prostate cancer. One group has investigated inhibition of PABPC1 in the context of pain reception and found that modified poly (A) RNAs can function as a decoy to trap PABPC1, thus preventing it from carrying out its function in nociception (41). Further research is necessary to determine if a similar approach would change the ability of PABPC1 to promote ERG function in prostate cancer.

To our knowledge, this is the first report of PABPC1 playing a role in ETS transcription factor function. However, a role of PABPC1 has been previously reported in prostate cancer cells. PABPC1 can function to regulate the nuclear localization of AR, and knockdown of PABPC1 reduces AR-positive prostate cancer cell proliferation and transactivation of AR target genes (42). ERG can directly interact with AR and can bind to DNA cooperatively with AR (43). Therefore, it is possible that AR interacts with both ERG and PABPC1 within a higher-order transcriptional complex at some genomic binding sites.

We found that PABPC1 directly interacted with the oncogenic ETS factors ERG, ETV4, and ETV5, but does not directly interact with ETV1. Interestingly, this is the same pattern of direct interactions between these ETS factors and EWS (8). Therefore, PABPC1 is not the factor that mediates the indirect interaction between ETV1 and EWS. Despite the similar pattern of interaction, the direct interactions between ETS and EWS are distinct from the direct interactions between ETS and PABPC1, as the former, but not the latter are disrupted by a P436A mutation in ERG. Additional work is needed to determine if PABPC1 indirectly interacts with ETV1 as seen with EWS, as well as the extent to which PABPC1 regulates the activity of the PEA3 subfamily (ETV1, ETV4, and ETV5) of ETS transcription factors.

PABPC1 appears to be a critical component of a higher order, nuclear complex involving ETS family transcription factors and EWS, that functions to activate transcription of target genes. As such, PABPC1 may be a viable therapeutic target for ETS-positive prostate cancer.

## Experimental procedures

### Cell culture and viral transduction

Cell lines were purchased from American Type Culture Collection and have been authenticated using the PowerPlex 16HS short tandem repeat profiling assay. Cells were tested for *mycoplasma* and were *mycoplasma* free. RWPE cells were grown in keratinocyte serum-free media (Gibco). VCaP cells were maintained in Dulbecco's modified Eagle's media supplemented with 10% fetal bovine serum (Sigma). PC3 cells were maintained in F12K media supplemented with 10% fetal bovine serum. All cells were incubated at 37 °C and 5% CO_2_, and all growth media supplemented with 1× penicillin/streptomycin (Corning). All shRNAs were packaged in lentiviruses produced by cotransfecting HEK-293T cells with pMDLg/pRRE (Addgene plasmid 12251), pRSV-Rev (Addgene plasmid 12253), and pMD2.G (Addgene plasmid 12259), packaging plasmids in addition to the pLKO.1 cloning vector (Addgene plasmid 8453) with the corresponding shRNA sequence. Oligonucleotide sequences are referenced in Table S1. Retroviral overexpression of ERG, ETV1, ETV4, EWS, ctEWS, and ntEWS were created using the method described in (8).

### Migration assays

The *trans-*well migration assay was previously described in (5). In short, Boyden chambers (8 μM pore size; BD Bioscience) were placed in the wells of a 24-well plate filled with 750 μl of serum-containing media. 5 × 10^4^ cells suspended in 500 μl of serum-free media were then plated into a Boyden chamber, and the cells were incubated at 37 °C for 72 h for RWPE cells or 48 h for PC3 cells. The media was then aspirated from the Boyden chambers, the internal portion of the membrane was washed with PBS and cotton swabs, and the membrane was stained with Hema 3 staining kit. The membranes dried for 24 h before being plated on microscope slides. Each condition was performed in duplicate, five images were taken per membrane, and cells were counted.

### Clonogenic survival assay

One thousand cells were plated in 3 ml of media per well of a 6-well plate. The cells incubated for 24 h before drug treatment. The plates were then incubated for another 9 days before the cells were fixed with 10% formalin and stained with 0.5% crystal violet in 25% methanol. The plates dried, and the colonies were imaged and counted with the Genesys software (www.syngene.com/software/genesys-rapid-gel-image-capture/). Each value reported is the mean of three biological replicates, each derived from the mean of three technical replicates.

### Immunoblots

Whole-cell extracts were collected with Nonidet P-40 (NP-40) lysis buffer (50 mM Tris–HCl pH 7.4, 250 mM NaCl, 5 mM EDTA, 50 mM NaF, and 1% NP-40). Extracts were separated on 10% SDS-PAGE gels and transferred to nitrocellulose membranes (Bio-Rad) using standard procedures. Membranes were blocked in 5% milk in tris buffered saline (10 mM Tris, pH 8, 150 mM NaCl), incubated with primary and secondary horseradish peroxidase–conjugated antibodies, and exposed to Super Signal enhanced chemiluminescence (Thermo Fisher Scientific). Antibodies used in this study are HA (H3663, Sigma), GST (NB600-326, Novus), ERG (CM421C, BioCare), His (2365S, Cell Signaling), ER81 (SC-1953, Santa Cruz), ETV4 (ARP32263-P050), FLAG (F1804, Sigma), Tubulin (T9026, Sigma), EWS (SC-28327, Santa Cruz), V5 (D3H8Q, Cell Signaling), and PABPC1 (SC-166027, Santa Cruz).

### Protein purification

Proteins were purified as previously described in (8, 44). His-tagged proteins were expressed from the pET28a vector (Novagen) in *Escherichia coli* BL21 cells induced with IPTG (0.5 mM) and were purified with nickel-nitrilotriacetic acid beads (Qiagen). GST-tagged proteins were expressed from the pGEX-6p-2 vector in *E. coli* BL21 cells induced with IPTG (0.5 mM). The proteins were purified from the cell lysates with magnetic GSH beads (Pierce) and eluted with GSH elution buffer (125 mM Tris, pH 8, 50 mM reduced GSH, and 150 mM NaCl).

### Affinity pulldowns

These were performed as described previously in (27). Briefly, 5 μg of purified His-tagged ETS proteins were bound to 2.5 μl His isolation magnetic Dynabeads (Thermo Fisher Scientific) in 300 μl of 2× binding buffer (100 mM sodium phosphate pH 8, 600 mM NaCl, and 0.02% Tween) for 1 h. The protein-bound beads were then washed twice with 700 μl of 2× binding buffer for 5 min per wash. The final wash buffer was removed and replaced with 500 μl NP-40 lysis buffer (50 mM Tris–Hcl pH 7.4, 250 mM NaCl, 5 mM EDTA, 50 mM NaF, and 1% NP-40) and 100 μg of bovine serum albumin (BSA) to block the beads of nonspecific binding. Then, purified protein or whole cell extracts were added to the beads and allowed to incubate at 4 °C for 2 h before triple washing with NP-40 lysis buffer. Beads were then resuspended in 20 μl of 4× SDS loading dye and boiled for 10 min before being loaded onto protein gels. Bead-bound protein was detected *via* either Ponceau staining (0.1% Ponceau stain in 5% acetic acid) or Western blot.

### Nuclear/cytoplasmic fractionations

Cells were scraped and harvested in 2.5 ml PBS (137 mM NaCl, 2.7 mM KCl, 10 mM Na_2_HPO_4_, 1.8 mM KH_2_PO_4_) and centrifuged at 400*g* for 5 min. The pellet was resuspended in 1 ml CEBN buffer (10 mM Hepes pH 7.8, 10 mM KCl, 1.5 mM MgCl_2,_ 340 mM sucrose, 0.4% NP-40) and rotated at 4 °C for 5 min. Samples were centrifuged at 1600*g* at 4 °C in a microcentrifuge, and the supernatant was retained separately as the cytoplasmic fraction, and the nuclear pellet was washed twice with 500 μl CEB buffer (10 mM Hepes pH 7.8, 10 mM KCl, 1.5 mM MgCl_2_, 340 mM sucrose). Washed nuclear pellets were then resuspended in 1 ml modified radioimmunoprecipitation assay buffer (50 mM Tris pH 7.5, 100 mM NaCl, 3 mM EDTA, 0.5% NP-40, 50 mM NaF). Cytoplasmic and nuclear fractions were sonicated for two 20 s cycles using a probe sonicator. Samples were centrifuged at 16,000*g* for 10 min at 4 °C, and the supernatant was retained to remove cellular debris.

### Densitometry analysis

Densitometry was performed in ImageJ (https://imagej.net). Plots of immunoblot intensity were generated, and the area under the curve was calculated. Values were then normalized to the area under the curve corresponding to the appropriate tubulin blots.

### Coimmunoprecipitation

Magnetic Dynabeads were combined with the appropriate antibody and 250 μl of NP-40 lysis buffer (50 mM Tris–Hcl pH 7.4, 250 mM NaCl, 5 mM EDTA, 50 mM NaF, and 1% NP-40) in an Eppendorf tube and rotated at 4 °C overnight. Cells were harvested from 15 cm plates with NP-40 lysis buffer and sonicated for two 20 s cycles using a probe sonicator. Debris was removed in a microcentrifuge at 15,000 rpm for 10 min. Equal amount of protein was added to each tube, and 5% of each sample was retained as input controls. Tubes were rotated at 4 °C for 4 h. The beads were washed four times with NP-40 lysis buffer for 5 min per wash. Samples were then resuspended in 4× SDS loading dye and loaded onto a gel to be run as a Western described above.

### RNA quantification

Cell lysates were homogenized with QIA-shredder columns (Qiagen), and RNA was extracted using the RNeasy Kit (Qiagen). RNA was quantified *via* the Nanodrop 2000c (Thermo Fisher Scientific). One percent β-mercaptoethanol was added to the RLT lysis buffer. RNA was quantified by reverse transcription and qPCR as described in (5). Reverse transcription reactions contained 500 ng of RNA, 500 μM dNTPs, 100 nM oligo primers, 1 × First Strand Buffer (New England BioLabs), 5 mM DTT (Invitrogen), 40 U murine RNase inhibitor, and 200 U Superscript III reverse transcriptase in 20 μl of total reaction volume. Reactions were incubated at 55 °C for 55 min, followed by 15 min at 70 °C. Five units of RNase H was added to each reaction and incubated at 37 °C for 20 min to degrade the remaining RNA templates. Complementary DNA was stored at −20 °C. Reactions for qPCR contained 1 × KAPA SybrFast qPCR master mix (2.6 mM MgCl_2_), 2 μl of complementary DNA or standard curve DNA, 2 μl DNase-free water, and 500 nM primers in a total reaction volume of 10 μl. Two technical replicates of each sample were plated in 96-well plates (VWR #83009-676) and read by a QuantStudio 3 thermocycler, and the data were analyzed using the Thermo Fisher Connect Design and Analysis 2 qPCR application (Thermo Fisher Scientific). A standard curve was generated for each target by running five 10-fold serial dilutions of standards. The PCR program was 95 °C for 20 s, followed by 40 cycles of 95 °C for 1 s and 60 °C for 20 s. Upon completion of the PCR, a melting curve was generated to validate specificity. RNA levels were normalized to 18S. The DNA oligonucleotide primers used were produced by Integrated Data Technologies and reported in Table S1. Primer specificity was screened *in silico* with UCSC’s BLAT tool.

### ChIP-seq and analysis

ChIP was performed as previously described in (5). Cells were cross linked with 1% formaldehyde (Thermo Fisher Scientific) for 15 min before being quenched with 2 M glycine for 5 min. The cells were then washed, lysed, and sonicated (Diagenode, Bioruptor Pico) at 4 °C for three cycles of 30 s on followed by 30 s off. The nuclear fraction was incubated with a PABPC1 antibody (SC-166027, Santa Cruz) conjugated to magnetic beads (mouse Dynabeads, Thermo Fisher Scientific) for 4 h at 4 °C. The beads were washed, and the DNA was isolated by a phenol/chloroform extraction. Libraries were performed for sequencing as previously described in (45). In summary, three ChIP experiments were pooled and sheared using a waterbath sonicator for ten cycles of 30 s on followed by 30 s off (Diagenode, Biorupter Pico). Illumina TruSeq barcode adapters were ligated to repaired ends of sheared fragments. Adapter-ligated fragments were PCR amplified and purified with Ampure XP beads and size selection from a 2% agarose gel to remove adapter dimer contamination. Libraries were pooled and single-end sequenced on an Illumina NextSeq 500 with a NextSeq75 SE. Reads were aligned to the human reference genome hg19 using Bowtie2. Duplicates were removed using samtools, and blacklisted regions were removed using bedtools. Peaks were called using MACS2, with a *p*-value cut-off of 0.001. Motif discovery was performed with RSAT (http://rsat.sb-roscoff.fr/), neighboring genes were determined with USeq FindNeighboringGenes tool, and gene ontology analysis was performed using Metascape (metascape.org) and CHIP-ENRICH (chip-enrich.med.umich.edu/). Metagene plots were created using the NGSPLOT package.

### Luciferase reporter assays

Cells were transiently transfected with 1 μg of renilla reporter and luciferase reporter using *Trans*-IT 2020. The luciferase reporter contains seven repeats of the ETS motif, GGAA, which was cloned into the firefly luciferase plasmid pGL4.25. Twenty four hours after transfection, cell lysates were collected and analyzed using Promega’s Dual Reporter Luciferase Assay kit. Briefly, cells were harvested with 250 μl of 1× passive lysis buffer, and the cells were lysed by undergoing four freeze-thaw cycles in liquid nitrogen. Lysate was then plated in Greiner Cellstar 96-well plates, Luciferase Assay Reagent II was added, and the luminescence was measured. Stop and Glo was then added to quench the firefly luciferase signal and activate the renilla luciferase. The renilla luminescence was measured and values were normalized to renilla signal.

### Immunofluorescence

One thousand cells were plated onto coverslips in 3 ml of media per well of a 6-well plate. Following 24 h incubation, cells were fixed in 4% paraformaldehyde (20 min), permeabilized with 0.2% Triton X-100 (5 min) and blocked for 1 h at room temperature in PBS (pH 7.4) containing 0.1% Tween-20 and 1% BSA. CoraLite 594–conjugated PABPC1 antibody (Proteintech Cat# CL594-66809; 1:200) in PBS containing 0.1% Tween-20 and 1% BSA were added overnight at 4 °C in a humidified chamber. Coverslips were mounted onto glass slides using VECTASHIELD mounting media with 4′,6-diamidino-2-phenylindole (Vector Laboratories H-1200) and sealed with nail polish to prevent drying and then stored at 4 °C. Images were obtained using fluorescence microscopy (Nikon Eclipse Ni-E) at magnification, 200×.

## Data availability

ChIP-seq datasets are available in the Gene Expression Omnibus (https://www.ncbi.nlm.nih.gov/geo/) under accession number GSE226472.

## Supporting information

This article contains supporting information.

## Conflict of interest

The authors declare that they have no conflicts of interest with the contents of this article.
