# Supplementary material for: A complex with poly(A)-binding protein and EWS facilitates the transcriptional function of oncogenic ETS transcription factors in prostate cells
Source: J Biol Chem. 2023 Nov 11;299(12):105453. doi: 10.1016/j.jbc.2023.105453 (PMC10704431; doi:10.1016/j.jbc.2023.105453)
Supplement: Supplemental Figures S1–S3 [file mmc1.pdf]

Figure S1

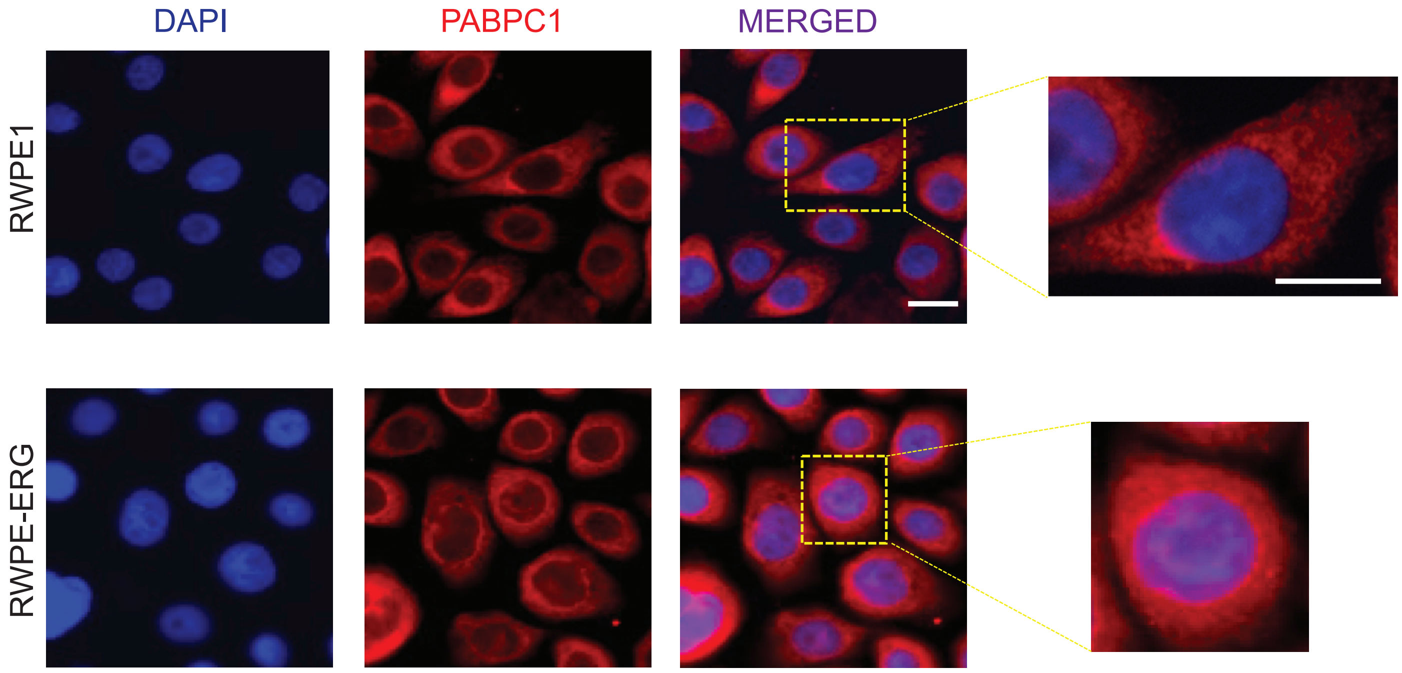

Figure S1. Representative immunofluorescence image of RWPE-1 and RWPE-ERG cells (grown on coverslips, stained with CoraLite 594-conjugated PABP1C antibody (red) and DAPI (blue). Images were obtained using fluorescence microscopy. Scale bar is 10 μm.

Figure S2

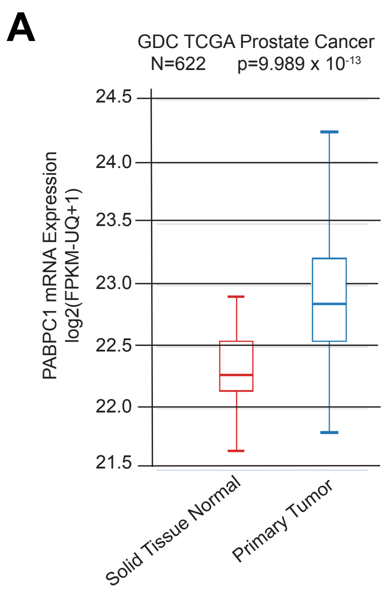

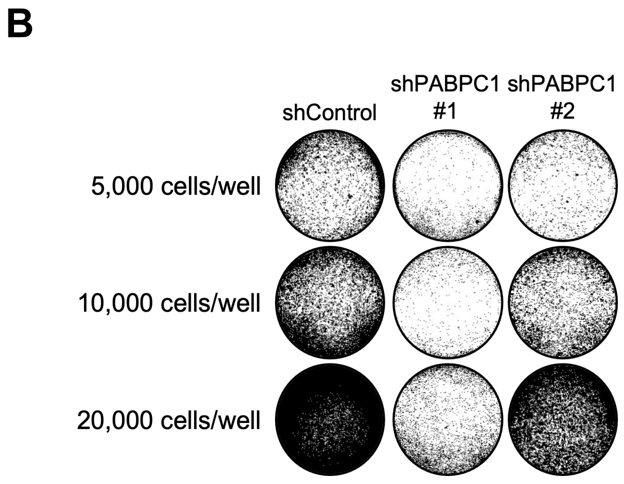

Figure S2. (A) PABPC1 mRNA expression levels from TCGA prostate cancer dataset. (B) Representative images of colony formation assay with VCaP cells. 10,000 cells per well were used for replicates quantified in Figure 5E.

Figure S3

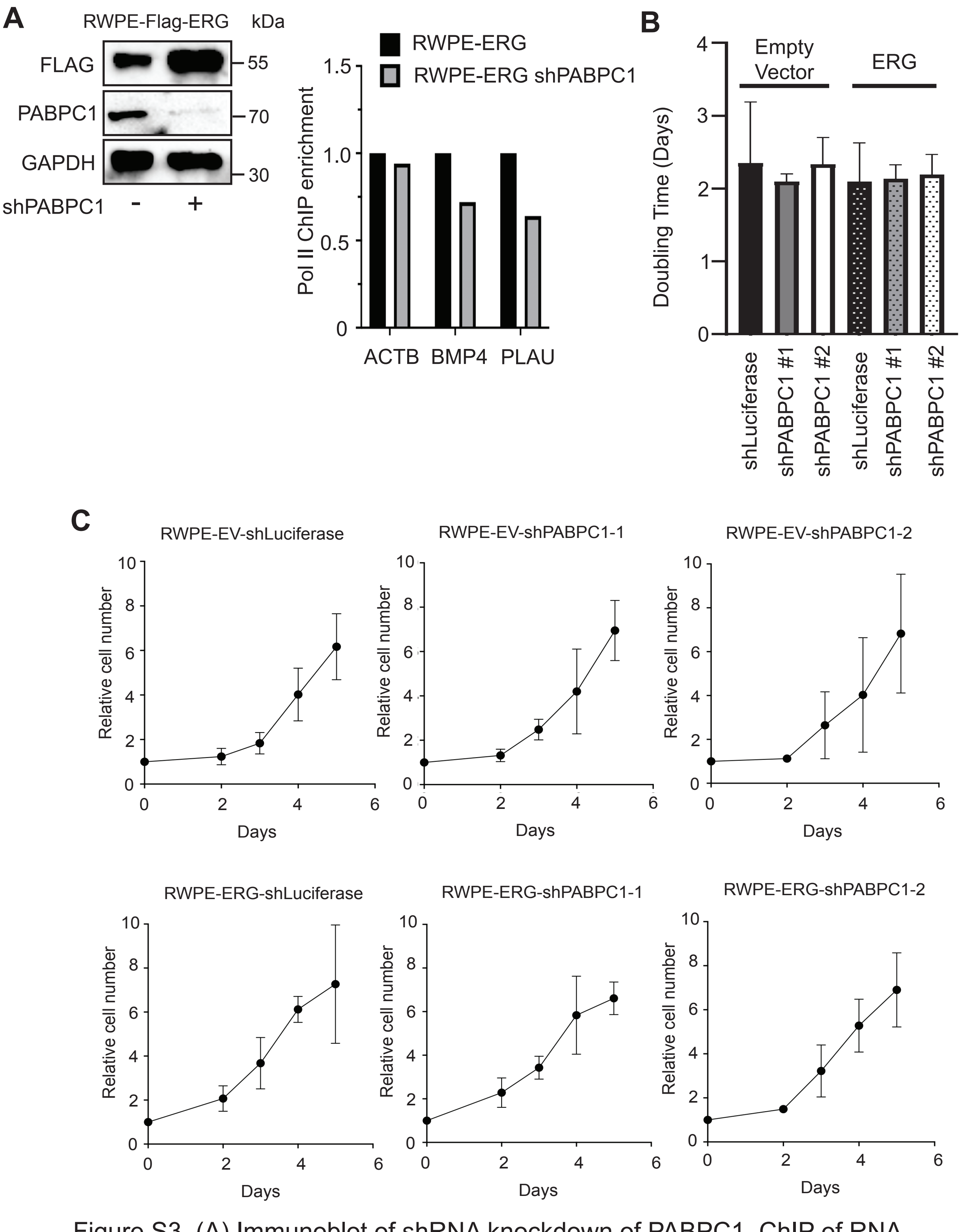

Figure S3. (A) Immunoblot of shRNA knockdown of PABPC1. ChIP of RNA polymerase 2 measured by qPCR of amplicons in the body of a non-ERG target gene, ACTB, and two ERG target genes, BMP4 and PLAU, all normalized to qPCR of a negative control region of the genome, then normalized to mock knockdown cells. (B,C) Growth of indicated cell lines was measured over 5 days by counting cell numbers in replicate wells. Doubling time (B) was calculated by the slope of the growth curves (C).
