# Supplementary material for: A complex with poly(A)-binding protein and EWS facilitates the transcriptional function of oncogenic ETS transcription factors in prostate cells
Source: J Biol Chem. 2023 Nov 11;299(12):105453. doi: 10.1016/j.jbc.2023.105453 (PMC10704431; doi:10.1016/j.jbc.2023.105453)
Supplement: Supplemental Table S1 [file mmc2.docx]

**Supplemental Table 1. DNA oligonucleotide sequences.**

| Oligonucleotide name | Sequence |
| --- | --- |
| PABPC1 shRNA #1 F | ccggaattgatcagggaccatgaaactcgagtttcatggtccctgatcaatttttttg |
| PABPC1 shRNA #1 R | aattcaaaaaaattgatcagggaccatgaaactcgagtttcatggtccctgatcaatt |
| PABPC1 shRNA #2 F | ccggaatagctcagaaatgtcagttcctcgaggaactgcacattctgagctatttttttg |
| PABPC1 shRNA #2 R | aattcaaaaaaatagctcagaatgtcagttcctcgaggaactgacattctgagctatt |
| 18S RT-qPCR F | ggtgaaattcttggaccggc |
| 18S RT-qPCR R | gactttggtttcccggaag |
| BMP4 RT-qPCR F | tacatgcgggatctttaccgg |
| BMP4 RT-qPCR R | tctgcagaggagatcacctcg |
| TGFBR2 RT-qPCR F | ggaagtctgtgtggctgtatg |
| TGFBR2 RT-qPCR R | tgcactcatcagagctacagg |
| PLAU RT-qPCR F | gctgacacgcttgctcacc |
| PLAU RT-qPCR R  ACTB ChIP-qPCR F  ACTB ChIP-qPCR R  BMP4 ChIP-qPCR F  BMP4 ChIP-qPCR R  PLAU ChIP-qPCR F  PLAU ChIP-qPCR R  Negative control ChIP-qPCR F  Negative control ChIP-qPCR R | cgttatacatcgagggcaggc  ggcatgggtcagaaggatt  gtgtggtgccagattttctc  atcacacgactactggacac  aggtaacgatcggctaatcc  gtcgctcaaggcttaactcc  cattatggccacagtagtcg  cagtgcacttgttgagctcg  ctccttatcgtcagccttgc |
